# Low-Voltage DC-DC Converter for IoT and On-Chip Energy Harvester Applications

**DOI:** 10.3390/s21175721

**Published:** 2021-08-25

**Authors:** Miroslav Potocny, Martin Kovac, Daniel Arbet, Michal Sovcik, Lukas Nagy, Viera Stopjakova, Richard Ravasz

**Affiliations:** Institute of Electronics and Photonics, Faculty of Electrical Engineering and Information Technology, Slovak University of Technology, Ilkovicova 3, 81219 Bratislava, Slovakia; miroslav.potocny@stuba.sk (M.P.); daniel.arbet@stuba.sk (D.A.); michal.sovcik@stuba.sk (M.S.); lukas.nagy@stuba.sk (L.N.); viera.stopjakova@stuba.sk (V.S.); richard.ravasz@stuba.sk (R.R.)

**Keywords:** DC-DC converter, charge pump, energy harvesting, low-voltage, IoT, wireless power transfer system

## Abstract

The power saving issue and clean energy harvesting for wireless and cost-affordable electronics (e.g., IoT applications, sensor nodes or medical implants), have recently become attractive research topics. With this in mind, the paper addresses one of the most important parts of the energy conversion system chain – the power management unit. The core of such a unit will be formed by an inductorless, low-voltage DC-DC converter based on the cross-coupled dynamic-threshold charge pump topology. The charge pump utilizes a power-efficient ON/OFF regulation feedback loop, specially designed for strict low-voltage start-up conditions by a driver booster. Taken together, they serve as the masters to control the charge pump output (up to 600 mV), depending on the voltage value produced by a renewable energy source available in the environment. The low-power feature is also ensured by a careful design of the hysteresis-based bulk-driven comparator and fully integrated switched-capacitor voltage divider, omitting the static power consumption. The presented converter can also employ the on-chip RF-based energy harvester for use in a wireless power transfer system.

## 1. Introduction and Motivation

Recent advances in design and fabrication of integrated circuits (ICs) opened a space for the development of wireless, miniature and powerful sensor nodes, as well as wearable biomedical devices. Since the device’s case size is constrained to a few cm${}^{3}$, standard IC power supplies using a battery are rather limited. This constraint forces the development of new IC design and power management methods for low-voltage and low-power conditions. These approaches include the following:

- Energy harvesting using power tracking algorithms;
- Low-voltage circuit topologies;
- Voltage converters with low start-up voltage;
- Low-voltage driving circuits.

Energy harvesters (EHs) are based on converting an alternative energy source to electrical energy [1]. Among others, photovoltaic, thermoelectric [2], piezoelectric [3,4], acoustic [5], triboelectric [6] or electromagnetic generators [7,8] are widely used for this purpose. Supplying the IC by using an appropriate EH allows the whole device to become energetically autonomous. On the other hand, considering a small build volume, the EH output voltage value and also power are insufficient in continuously supplying an integrated circuit. As overall EH methodology has become promising, solutions for the above mentioned issue have emerged.

The radio-frequency energy as a potential power source reaches relatively low power density compared to solar and piezoelectric energy [9]. On the other hand, it is more stable due to source regularity and lower weather dependency. Taking these attributes into account, the radio-frequency energy harvesting (RF-EH) is widely adopted for ultra-low power IC and their application. The RF-EH achieves high reliability and small form factor of a transducer. According to the state-of-the art, the RF-EH systems dominantly employ inductive DC-DC converters. These are more attractive compared to the capacitive counterparts (charge pumps) due to the higher power conversion efficiency (PCE) and suitability for low-voltage start-up [10]. Recent research has revealed that the charge pump-based step-up converters reach improved PCE and start-up ability [11,12]. However, the research across the utilization of charge pumps (CPs) in energy harvesting systems is relatively unexplored [13,14]. This inspires further research towards the examination of CPs as an alternative to inductive step-up converters for ultra-low voltage EH systems.

As the output impedance of an EH system is rather high, it is essential to match the following IC input impedance in order to preserve the EH source capability. The more sophisticated approach is the use of the maximum power point tracking (MPPT) algorithms [15]. MPPT dynamically adjusts the EH load impedance according to its power curve, allowing higher power extraction. In order to achieve sufficiently high value of supply voltage for IC using EHs, the switched regulators are used to carry out the conversion of voltage from lower to higher values. One of the widely used regulator topologies suitable for full integration on the chip are CPs. Its operation is based on a charge being accumulated in capacitors and sequentially propagated to the output through switches. As the charge pump itself must be designed for ultra-low voltage conditions, while being supplied from an EH, the proper operation of switches becomes a challenge. For charge accumulation, CPs utilize rather large capacitors. Such capacitors require adequate drivers, which are well suited with a simple inverter. In order to maintain sufficient propagation delay during capacitor charging, which limits the overall operation, it is crucial to open and close the inverter transistors as much as possible. This otherwise trivial requirement becomes a challenge in the environment of supply voltage under 200 mV. Therefore, the clock signals (CLKs) require a dedicated boosting circuit with adequate driving capability, increasing the value of CLK signal [16]. As every voltage conversion technique has its limits from the efficiency point of view, it is essential to design ICs using low-voltage and low-power approach. In the field of analog IC design, such methods include the $g_{m}/I_{D}$ design methodology and the so-called bulk-driven approach. Such methods of IC design for ultra low-voltage applications form the core of the research presented in this paper, where the bulk-driven design approach has been implemented in the design of several subcircuits (e.g., a charge pump core, a boosted driver, comparator and a voltage divider) in order to improve the total EH performance focused on low-voltage conditions.

From the state of the art development point of view, the most important attributes of the developed EH power management unit (EH-PMU) based on the proposed DC-DC converter are the following:

- Minimum start-up voltage of CP;
- Output-to-input voltage ratio;
- Output voltage ripple;
- Self-sustainability;
- Power throughput;
- Overall EH-PMU effectivity in the target power range.

The last three attributes represent the primary objectives for our research, where the self-sustainability and the overall EH-PMU efficiency are ensured by low-power requirements originating from analogless design, and the power throughput is achieved by the modified structure of the CP core using a dedicated driver and taking into account reliable switching and the associated frequency/timing requirements. The CP-based PMU functionality has been verified by experimental measurements and was successfully implemented into RF-EH systems. Moreover, the valuable experimental results from the bulk-driven design represent a part of the novelty in terms of the application of unconventional design approaches in low-voltage systems.

The paper is organized as follows. In Section 2, the developed DC-DC converter and its main building blocks are described. Section 3 presents the application of the converter in an RF-based energy harvesting system. Evaluation results obtained by the measurement of the experimental chip prototypes are presented in Section 4, where simulated and measured results are compared as well. Comparison of the results achieved in this research with other works is addressed in Section 5, where a short discussion and conclusions can also be found.

## 2. DC-DC Converter System

**Figure 1:**
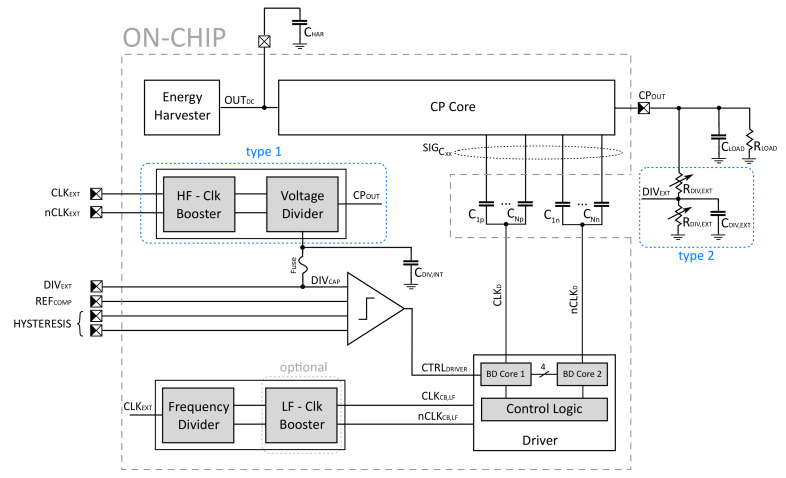
Top level of the proposed CP-based DC-DC converter.

In order to address the issues and trends described in the previous section, a regulated low-voltage (subthreshold) step-up DC-DC converter based on switching capacitors, also known as a charge pump, has been proposed. Figure 1 shows the top level block diagram of the developed system with highlighted parts that were integrated on a chip. The CP-based converter consists of the following parts: a CP Core as the main part of the voltage stepping and conversion, an integrated voltage divider realized by switching capacitors (marked as type 1), clock boosters driving digital logic blocks with potentially mismatched supply voltages, a low-voltage comparator, a CP driver and a frequency divider with fixed dead time. The proper connection of these parts into a feedback configuration can control the output voltage $V_{CP_{OUT}}$ to the desired regulated value according to custom specifications. The ON/OFF based regulation feedback loop approach is implemented to manage this task.

### 2.1. Charge Pump Core

**Figure 2:**
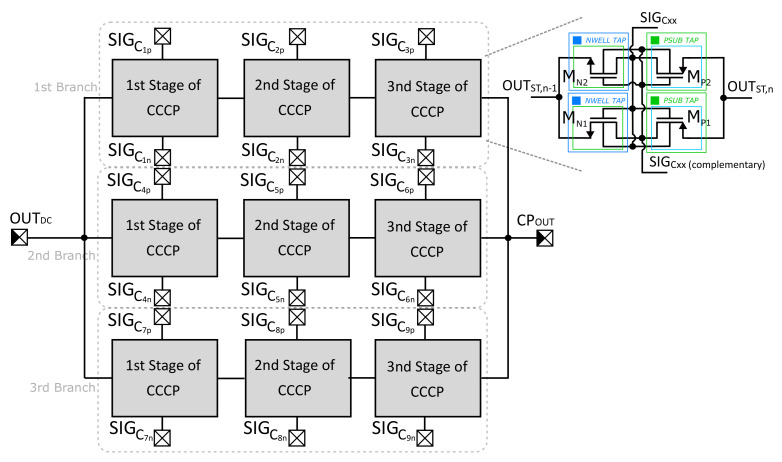
Multi-stage CP Core block (detail view of the DT-CCCP cell).

The most important part of the proposed converter is the CP Core (Figure 2). This block consists of nine cells organized into series-parallel configuration creating an unconfigurable structure with three parallel branches (multi-branch structure at higher system level). Each branch consists of three CP cells connected into a cascade. In the ideal case, cascading cells allows boosting the CP Core input voltage $V_{OUT_{DC}}$ up to $V_{OUT_{DC}}+NV_{OUT_{DC}}$, where N is the number of cascaded stages. This can be achieved if supplying the CP core as well as the driver by the same voltage value. However, non-ideal CPs have many parasitics that deteriorate performance such as the finite output impedance including modulation index and parasitics capacity at switching nodes, etc. [17,18,19]. Under low-voltage subthreshold conditions and the high current throughput requirement (see RF-harvester application in Section 3) across the system, the output impedance plays a very important role and should be minimized as much as possible. In this sense, we would like to state the following:

(a) *Impedance modulation index*, which is the result of finite on-state transistor resistance $R_{T,ON}$ of main switches—$M_{N1-2}$ and $M_{P1-2}$ (see Figure 2) – is usually reduced by bootstrapping techniques implemented in the CP cell [20,21];
(b) The ideal term $\propto N_{C}/kF_{SW}C_{FLY}$, which is valid for linear CP, is topology-dependent and can be managed exclusively by topology interventions. $N_{C}$ is a number of stages in the cascade, *k* is a number of power flow patches known as branches, $F_{SW}$ is clock frequency and $C_{FLY}$ represents the main flying capacitor (in Figure 2 denoted as $C_{1x-Nx}$, where *x* stands for *p* or *n*) [18].

In order to deal with statement (a), the Dynamic Threshold Cross-Coupled Charge Pump (DT-CCCP) has been chosen as a good candidate. Generally, the CCCP has promising potential under low-voltage conditions without applying any special "external" techniques since bootstrapping intrinsically includes such a feature. Despite the few inherited drawbacks, the positives outweigh negatives from a broader perspective [22,23,24]. Moreover, recent research proves that if the dynamic-$V_{TH}$ technique is employed to minimize the impedance modulation index, together with careful design based on a parasitics-aware approach, the current throughput and power efficiency can be very optimistic even in the case of the full on-chip implementation [17]. On the other hand, in relation to statement (b), the CP topology intervention is required. Taking into account the current throughput requirement, the linear dual-branch CP has been selected because it is characterized by lower sensitivity of voltage conversion to the presence of parasitics capacitance (under the same capacitor requirements in the total value) compared to the non-linear CPs (as Fibonacci or exponential ones). For this reason, linear CPs are more suitable for heavy loads since a lower number of stages is needed for the same conversion ratio, and hence the lower output impedance [19]. Additionally, the branch parallelization (equivalent to multi-branch approach at CP cell level) at higher system levels has been used to redistribute the current and decrease the output impedance in both terms (a) and (b) (the value of the individual capacitors was retained).

Transformation of a single branch into multiple ones withdrew the need for on-state resistance $R_{T,ON}$ of the main switches $M_{N1-2}$ and $M_{P1-2}$ but brings more strict requirements for the driver, which charges flying capacitors $C_{1x-Nx}$ and shifts-up the voltage at their terminals. Since the system is synchronized into two clock phases, the driver’s inverter must sustain currents flowing through all capacitors during individual phases without any drastic impact on the impedance modulation index. It is important to note that if the system can be investigated through a representative cell [17], such a modification does not affect the ${\left(RC\right)}_{cell}$ constant of the charging/discharging cell itself if the values of transistor and capacitor are preserved. However, if a driver is non-ideal, i.e., on-state resistance $R_{D,ON}$ has a finite value, the original $R_{T,ON}C_{TOT}$ is increased about $NR_{D,ON}C_{TOT}$, where $C_{TOT}$ represents the total capacitance observed at the internal center node of the CP cell and *N* is the total number of CP stages.

### 2.2. Boosted Driver

**Figure 3:**
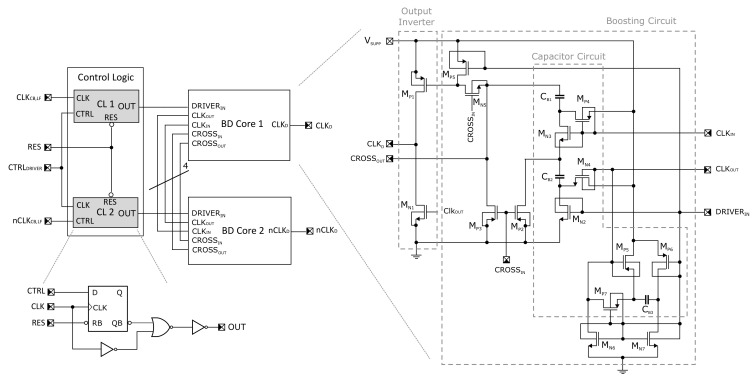
The driver scheme using bulk-driven cross-connection cores.

**Figure 4:**
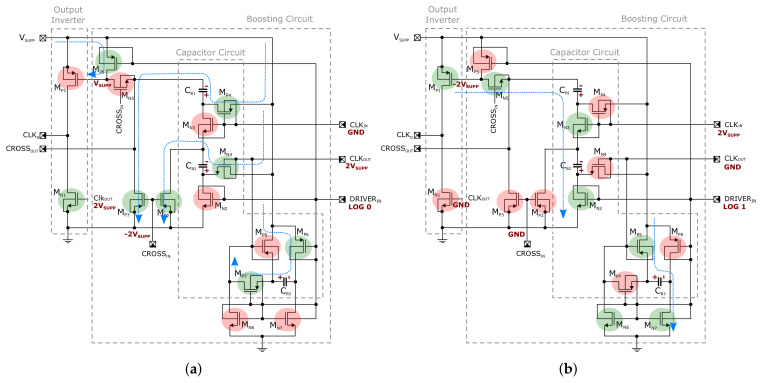
Driver operation principle. (**a**) $DRIVER_{IN}=Log\;0$. (**b**) $DRIVER_{IN}=Log\;1$.

Consequently, the proposed CP consists of a stand-alone boosted driver circuit to solve the above-mentioned issue as an alternative to other competitive techniques with different primary purposes in relation to ultra-low voltage research area (e.g., a clock booster based driver, topology reconfiguration approach, implementation of adiabatic principle and many others [10,24,25]. The transistor level schematics of the proposed driver and detailed cross-connection with a complementary replica circuit are shown in Figure 3 and Figure 4, respectively. The implemented driver originates from an already characterized version (presented in [26,27]), which was extended by another auxiliary circuit in order to further improve the performance of the boosted output inverter. The previous version boosts the gate of the high-side part of the inverter formed by PMOS transistor $M_{P1}$ to the negative voltage of −$2V_{SUPP}$. The low-side part was of our interest and, therefore, kept untouched. Despite this implementation, the driver has been successfully implemented into a chip prototype of regulated DC-DC converter and is capable in driving more than 100 pF capacitor with a few units of ns propagation delay under the supply voltage value of 200 mV and the clock frequency of 1 MHz [26].

In the converter design, the CP Core has been modified, including a larger number of stages. For this reason, the driver also had to be adapted to that and the improved version also takes care of the inverter low-side formed by the NMOS transistor $M_{N1}$ across the boosted gate voltage of $2V_{SUPP}$ in the ideal case. The operation principle can be closely examined from Figure 4, where the transistors and capacitors are organized in order to enable a serial-parallel reconfiguration for doubling the voltage value. In Figure 4, the states of individual transistor switches in both clock phases are highlighted, where green and red represents the on-state and off-state, respectively. The blue arrows show the energy flow of the charging/discharging process. It should be noted that the presence of positive voltage higher than the supply value, derived for the input voltage $V_{OUT_{DC}}$ in our case, is not only used for driving the main switch $M_{N1}$ but also controls the auxiliary NMOS switches. Including all logic gates, the dynamic-$V_{TH}$ technique has also been employed for some critical transistors in the driver, and for this reason natural trade-off between the maximum supply voltage, switching frequency and capacitor/transistor sizes exists. For instance, the node $CLK_{OUT}$ is relatively sensitive to this trade-off because, during time of driving transistor $M_{N2}$ to the voltage of $2V_{SUPP}$, the $M_{N2}$ transistor has a negative potential of about −$V_{SUPP}$ at its source terminal and the bulk diode can be activated to discharge $C_{B3}$ and charge $C_{B2}$ (both effects have negative impact on the driver performance). As a consequence, if $2V_{SUPP}$ represents a value that is too high and the frequency is too low, the driver can be the most critical loss contributor, and this is manifested in squeezing the efficiency for the higher input voltage compared to the lower one (in relation to the lower and upper switching frequency). Based on the simulations, in order to ensure a reliable functionality under the nominal conditions across all technology corners and the temperature range (−20 ∘C–85 ∘C), the size of capacitors $C_{B1}$ and $C_{B2}$ should be doubled (15 pF ⇒ 30 pF) compared to the previous version, and the value of the additional capacitor $C_{B3}$ has to set to 40 pF. It should be also noted that the performance of the proposed driver has not been explicitly investigated but was implicitly carried out only as a part of the system to which this work is primarily dedicated.

The inputs to the driver cores are generated by a simple logic acting as gated clock generator controlled by a comparator with hysteresis across a feedback loop. The frequencies of clocks, i.e., $CLK_{CB,LF}$ and complementary twin $nCLK_{CB,LF}$, are managed by an external $CLK_{EXT}$ signal with half of the clock frequency (see Figure 1). In other words, the frequency divider, with a non-overlapping clock generator, was implemented through standard T flip-flop topology followed by the NAND-based dead-time circuit (with delay gates in feedback path defined). Since the entire driver and frequency divider are supplied by the same voltage $V_{OUT_{DC}}$ during the experiment, using a clock booster or a level shifter in low-frequency branches is irrelevant. Therefore, an optional one (bounded by dashed gray-box in Figure 1) has been designed on a chip but omitted in measurements. We have to make it clear that in Section 4, the frequency range of 5–200 kHz representing the external clocks and clocks for switching the driver has to be considered as smaller by half.

### 2.3. Voltage Divider Branch

**Figure 5:**
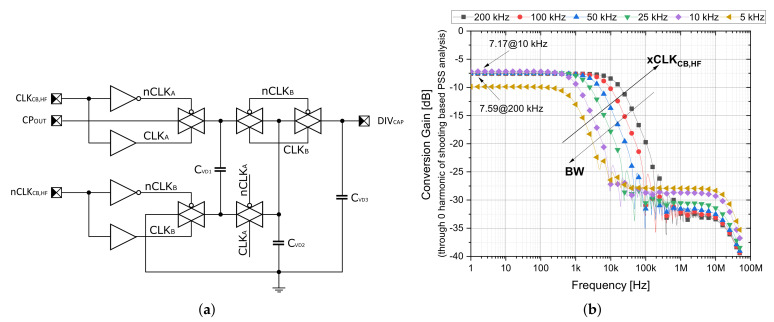
Switched-capacitor voltage divider 1 to 0.5. (**a**) Circuit implementation. (**b**) PEX simulation of conversion gain function under $V_{CP_{OUT}}=400\;mV$ and typical 23 ∘C conditions. Clocks and digital blocks are supplied by the same voltage.

The high-frequency branches are directly fed from the external clocks and drive transmission gates (TGs) in the capacitor-switching voltage divider across fast drivers and inverters (designed as *type 1* in top-level block scheme, Figure 1). The main objective of this step is to eliminate static power consumption and exploit a greater inherited compactness compared to bulky resistors with higher resistance value. The proposed voltage divider of *type 1* is shown in Figure 5a, where capacitors can be arranged by TGs connected into a serial-parallel cycling reconfiguration. Due to the fact that capacitors $C_{VD1}$ and $C_{VD2}$ are of the same size (capacitance of 15 pF), the ratio of ≈0.5 is provided at steady state. However, if a dynamic-$V_{TH}$ technique is employed together with quite large TGs ensuring reliable on-state under low-voltage conditions ($CP_{OUT}\;\approx$ 400 mV, $V_{TH}$ = 250–300 mV/300–350 mV for long/short channel, respectively, in a standard 130 nm CMOS technology), the dominant parasitics effect such as charge injection and clock feedthrough cannot be fully neglected. Additionally, the dynamic bulk-switching fosters discharging effects that degenerate the dividing ratio (the issues of low frequency). For these phenomena, the post-extract simulation (PEX) under the nominal conditions reveals 92–224 mV of $DIV_{CAP}$ variation in DC value, where for 25 kHz and 50 kHz driving frequencies, the best results of 193.4 mV and 210.3 mV are achieved, respectively. Figure 5b shows the conversion gain transfer function of the $CP_{OUT}$ to $DIV_{CAP}$ node through zero harmonic obtained from the PSS-PAC type analysis. The achieved results demonstrate slightly lower ratio around −7.4 dB in comparison to the intended value of −6 dB. With a link to time-based analysis observations, the non-negligible influence of parasitics effect can be deduced. Adding the filtrating capacitor $C_{VD3}$ with the capacitance value of 40 pF at the output node, the capacitor-switching voltage divider is obtaining a character of first order low-pass filter with frequency-dependent corner frequencies of the 1 kHz–20 kHz range related to clocks $CLK_{CB,HF}$ and $nCLK_{CB,HF}$, which is the characteristic property of many other systems utilizing the switching capacitor technique. Since the divider and control logic are necessarily supplied by the high-voltage domain (i.e., $V_{CP_{OUT}}$), the use of the clock booster or level shifter auxiliary circuits must also be a part of the design. In other words, the low-voltage domain (supplied by the input voltage $V_{OUT_{DC}}$) must be able to control the high-voltage domain and ensure its reliable operation. For this purpose, the popular Nakagome’s clock booster with dynamic-$V_{TH}$ technique was employed [20,28].

In order to create a complete picture about the developed CP system, it should be also noted that based on the observations described above, an alternative solution in the form of a common resistor-based voltage divider (marked as *type 2* in Figure 1) has been investigated, and a comparison is carried out in Section 4.1. In this case, the voltage divider has been realized externally (off-chip) and connected through the $DIV_{EXT}$ node. Thus, the supply voltage of the switched-capacitor voltage divider has been grounded, and the fuse has burnt out.

### 2.4. Comparator with Hysteresis

**Figure 6:**
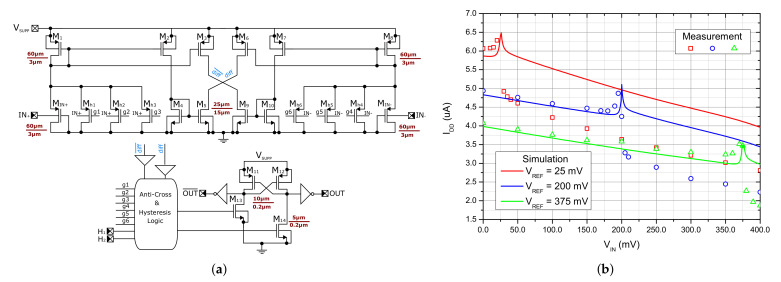
Implemented bulk-driven rail-to-rail comparator (redrawn from [30]). (**a**) Transistor level diagram. (**b**) Current consumption for various reference voltages.

The divided voltage $V_{DIV_{CAP}}$ is compared to the reference voltage $V_{REF_{COMP}}$ generated externally, and a suitable control signal at node $CTRL_{DRIVER}$ is produced (see Figure 1). The decision block providing this task is a rail-to-rail voltage comparator depicted in Figure 6, which has been designed for the power supply voltage of 400 mV with emphasis on the robustness and low value of the input offset without the need of post-processing trimming [29,30]. The topology is capable of working with even lower supply voltages thanks to two stacked transistors. The proposed comparator works in the so-called current regime. The input transistors act as current sources modulated by the input voltage applied to their bulk terminals. The current generated in the input branches is then mirrored in order to create the differential voltage in nodes diff and $\bar{diff}$, which is then processed by digital block. The proposed comparator topology also contains built-in programmable hysteresis controlled by a 2-bit input signal. The hysteresis does not require any external components and its level and symmetry can be fully custom-designed by the W/L ratio of the respective transistors. The enable function shuts-off the current sources, sets the output to a defined logic state, and reduces the power consumption into a leakage level. For this purpose, the Log0 level has been selected to allow passing the clock signals into the driver, which in this application ensure a reliable start also at a very low supply voltage. The selected measured characteristics are displayed in Figure 6b, where the measured current draw includes the consumption of several ESD structures, IC package, PCB and probe parasitics. This means that for on-chip application, the power consumption can be considered near or even less then 1 µW under typical conditions, which can be improved by further reduction of the supply voltage.

## 3. Application

In order to demonstrate the suitability of the developed DC-DC converter in the application example as a PMU in an RF-based energy harvesting system (RF-EH), we demonstrated its functionality in conjunction with a fully integrated wireless power transfer (WPT) receiver system. Thus, this section provides a brief description of the implemented fully integrated rectenna (rectifying antenna) based solution used for experimental validation. Because of the fabrication related limitations, a very low overall coupling coefficient is expected due to the maximum allowable area of the on-chip antenna (OCA), resulting in limited output power. Therefore, the internal components of the WPT system were optimized for low-voltage and low-power operation. Such a WPT system is used as a power source for the DC-DC converter and, therefore, is connected to the converter input (the Energy Harvester block in Figure 1). A more detailed description of the implemented WPT system can be found in [31].

### WPT System Description

**Figure 7:**
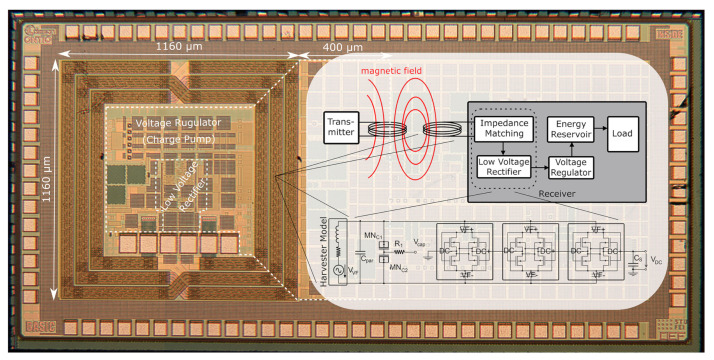
Microphotograph of the fabricated WPT chip. The $V_{DC}$ represents the $V_{OUT_{DC}}$ from Figure 1, i.e., the input voltage source for the CP.

A fully integrated WPT system can be divided into three main parts: the OCA, an impedance matching network and the rectifier circuit, as shown in Figure 7. The main design consideration of the latter two parts are the parameters of the OCA itself, as the antenna determines the usable frequency band as well as the available power of the system as a whole.

**Figure 8:**
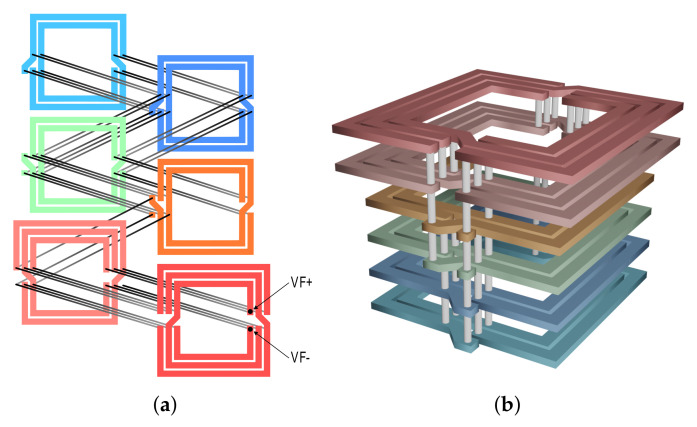
Designed OCA of the implemented EH (turn details: width = 60 µm, space = 12.5 µm; realized using top 6 layers. (**a**) Two-dimensional structure. (**b**) Three-dimensional structure.

The implemented OCA has been optimized for the limited available chip area on the manufactured prototype. In order to compensate this major limitation, a layer-modified version of the recently proposed structure (described in [32]) was used. The designed OCA structure is shown in Figure 8, where the topology is based on a symmetrical multi-layer stacked inductor suitable for use in a standard CMOS technology. In comparison to more commonly utilized designs, based on simple spiral coils, this proprietary structure offers improved inductance as well as quality factor values, both of which are very important parameters for the overall WPT system performance. Such an inductor design achieves the best overall performance at frequencies around 200 MHz ± 10%, with the previously measured example exhibiting the maximum quality factor in this region.

The impedance matching network and the main rectifier circuit of the proposed WPT system were designed to best utilize the above-described inductor design, taking into account the expected values of quality factor and inductance of the OCA. The impedance matching network was designed for the maximum power transfer, as the expected harvested values are very low due to the chip area restrictions. By utilizing a capacitor-only structure, we avoided possible interactions between multiple on-chip coils. The implemented structure consists of a tunable parallel capacitor used to form a resonant circuit with the EH coil and, thus, raise the harvested voltage and a series capacitance formed by the rectifier structure itself [33]. The core of the rectifier is formed by a differential drive cross-coupled (DDCC) CMOS bridge topology, which was previously found to be suitable for very low values of the input voltage and power [34,35,36]. The utilized circuit consists of three rectifier stages capacitively coupled to the EH coil. Each stage utilizes threshold voltage compensation for its internal switching transistors (based on the body-biasing technique) in order to improve the performance with the expected low RF input voltage amplitudes. The use of multiple stages was necessary to increase the total output DC voltage to a satisfactory value at the cost of somewhat decreased overall efficiency.

For the purpose of experimental verification, an external transmitter circuit consisting of a PCB coil and a lumped impedance matching network connected to a RF signal generator was designed. Two transmitter coil sizes were implemented based on sizing restrictions from [37,38]. These preliminary measurements were performed on bare die samples of the developed prototype chip, as presented in [39]. The measurements with the transmitter input power between 10 dBm and 20 dBm were performed with the maximum reported DC output power of around 60 µW and the maximum DC output voltage of more than 0.5 V under various load conditions. These results prove the overall functionality of the implemented WPT system, as well as its suitability for use in conjunction with the developed DC-DC converter described in the previous section.

## 4. Measurement and Achieved Results

In this section, the results obtained by the measurement of the prototyped chips are presented and discussed, where three branched configuration has been chosen for implementation as described in Section 2.1 in more detail. The selected configuration is capable to exploit the common power availability in 10–100 µW range of different energy harvester types used for IoT applications without significant form-factor violation, where the thermoelectric, bio-fuel cell or RF-based harvesters could be good candidates for utilization [1]. In order to better show the features of the proposed self-powered CP system, the measurements were performed without and with a RF energy harvester. For this purpose, the printed circuit test board was designed and fabricated. The measurement setup consists of the device under test (DUT) with all its physical peripheries and additional circuitry as well as the instrumental equipment where the input capacitor and the output capacitor placed directly on the test board possess a value of 35 µF and 69 µF, respectively. The flying capacitors belonging to the CP have the capacitance value of 330 nF and passive components corresponding to a voltage divider of *type 2* consisting of 2 × 600 kΩ resistors in series with a 8.5 pF capacitor at center node $DIV_{EXT}$.

### 4.1. Self-Powered CP System

**Figure 9:**
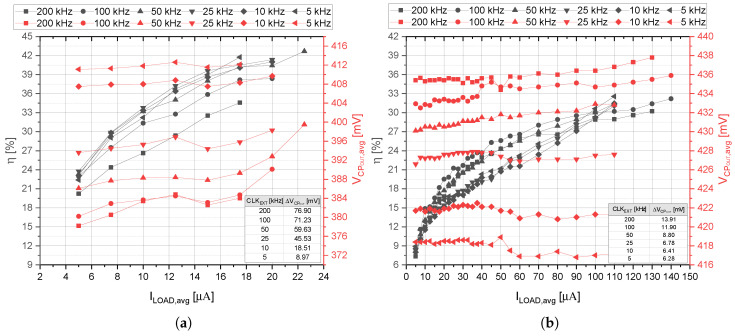
η and $V_{CP_{OUT}}$ vs. $I_{LOAD}$ for different clock frequencies (“avg” in graphs stands for average value). (**a**) For the $V_{DC_{IN}}$ of 180 mV. (**b**) For the $V_{DC_{IN}}$ of 300 mV.

In Figure 9a, one can observe the dependence of the efficiency (η) and the output voltage ($V_{CP_{OUT,avg}}$) of self-powered CP system on the output load current ($I_{LOAD,avg}$) for different external clock frequencies ($xCLK_{EXT}$) and the input voltage value $V_{OUT_{DC}}$ of 180 mV (the minimum value needed to start-up the self-powered CP system). It can be observed that the maximum efficiency can be obtained for a low clock frequency and for the maximum current at the CP output. For these conditions, the efficiency of about 42% can be achieved. However, the output voltage deviates by about 3.75% from the required value (400 mV). For higher clock frequencies, the CP system has a higher power consumption; therefore, the overall efficiency is degraded. The optimum clock frequency is about 50 kHz, where the efficiency and the output load current of 43% and 23 µA were achieved, respectively. As can be observed from Figure 9a, if the input voltage is increased to the value of 300 mV, the output current over 100 µA can be reached. In such a case, the efficiency of the self-powered CP system is less dependent on the clock frequency. It is also important to mention that the deviation of the output voltage of the CP system is mainly caused by the on-chip voltage divider, because its voltage ratio depends on the clock frequency as already discussed in Section 2.3.

**Figure 10:**
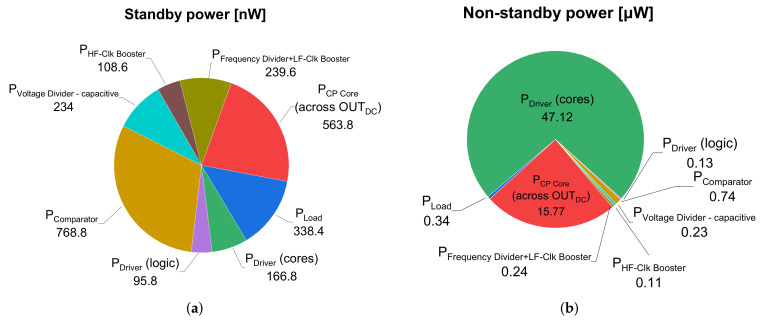
Distribution of power consumption across individual subsystems ($V_{DC_{IN}}=200\;mV$, $R_{LOAD}=476\;k\Omega$ and $xCLK_{EXT}=50\;kHz$ ). (**a**) Standby mode. (**b**) Non-standby mode (switching mode).

The stand-by power consumption and non-stand-by power consumption of the CP system are shown in Figure 10, where the total power consumption was divided into the contribution of individual sub-blocks of the overall CP system. Naturally, the biggest part of power consumption in the non-standby mode is given by CP Core and Driver cores that directly drive the bulky flying capacitors across charge transfer switches and boosted inverter, respectively. Other sub-blocks consume less than μW of total power consumption. In the standby mode, all parts have a power consumption in the order of hundreds nW, while the biggest part of contributors to standby power is given by the comparator used in the feedback control loop across its permanent current-bias and leakage current through bulk diodes (a natural drawback of bulk-driven design technique).

**Figure 11:**
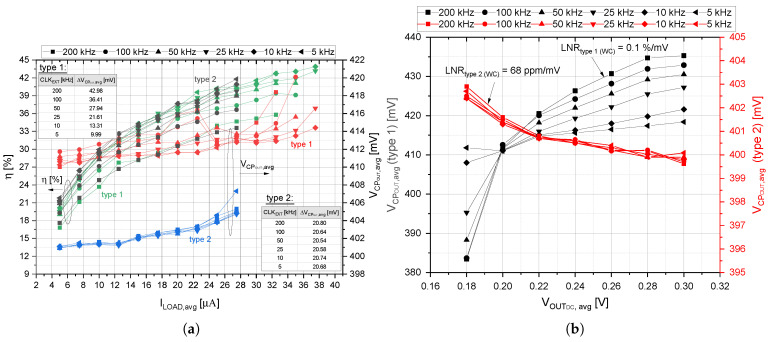
Comparison of different voltage dividers (“avg” in graphs stands for average value). (**a**) The η and $V_{CP_{OUT}}$ vs. $I_{LOAD}$ for $V_{DC_{IN}}$ of 200 mV. (**b**) The $V_{CP_{OUT}}$ vs. $V_{OUT_{DC}}$ for the $I_{LOAD}$ of 10 µA.

In order to investigate the possible influence on the CP system efficiency as well as for comparison of features and properties of the developed system, different types of voltage dividers were used in the measurements: (a) on-chip capacitor-based voltage divider and (b) off-chip resistive voltage divider, marked as *type 1* and *type 2* (Figure 1), respectively. As can be observed from Figure 11a, the off-chip resistive voltage divider (marked as type 2) can help in stabilizing the undesired output voltage deviation caused by different clock frequency. One can also notice that the output voltage ripple is relatively constant over the clock frequency. The efficiency of the self-powered CP system is not influenced by the type of the voltage divider used. However, the maximum load current and efficiency reached slightly higher values in the case of using the capacitor-based voltage divider (marked as *type 1* in Figure 11a.

Dependence of the output voltage on the input voltage is depicted in Figure 11b, where one can observe that the output voltage is independent of the clock frequency in the case of the resistive voltage divider. Additionally, the load regulation (LNR) in the worst case is 68 ppm/mV. Therefore, in order to obtain better output voltage regulation, it would be proper to use the resistive voltage divider. From the efficiency and the output load current point of view, the performed analysis show better performance in the case of using the capacitor-based voltage divider. However, such a divider should be carefully optimized for the selected clock frequency and parasitics effect in order to obtain desired output voltage regulation.

### 4.2. Self-Powered Cp System with the Rf Harvester

The results presented in the previous section were obtained with an independent voltage source used at the CP input node $OUT_{DC}$ (see Figure 1). In this section, we present the measurement results of RF-EH system as the input voltage source for the self-powered CP system is generated directly from WPT unit. The developed RF-EH was described in Section 3. The overall system was measured for different input power $P_{IN}$ of the external antenna and two distances between the external antenna and the implemented OCA (a part of the RF-EH).

**Figure 12:**
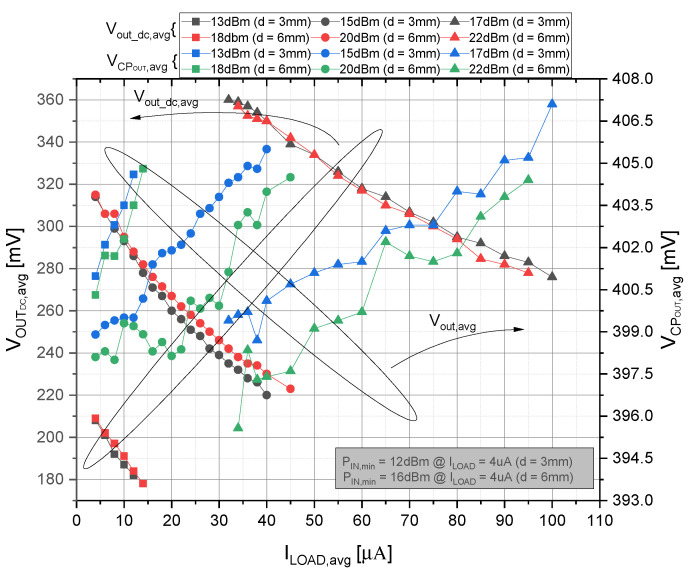
Individual output voltages $V_{OUT_{DC}}$ and $V_{CP_{OUT}}$ vs. $I_{LOAD}$ for different input powers (“avg” in graphs stands for average value). The $CLK_{EXT}$ is set to 50 kHz, and the voltage divider of *type 2* has been used.

Figure 12 shows the dependence of the CP output voltage (and the output voltage from the on-chip RF rectifier) on the load current at the output of CP system. The presented measurement results are obtained for the distance of 3 mm and 6 mm between the external antenna and the on-chip one for different values of the input power. The average output voltage from the rectifier is shown on the left y-axis ($V_{OUT_{DC},avg}$), while the regulated output voltage from the self-powered CP system is introduced on the right y-axis ($V_{CP_{OUT},avg}$) of the graph shown in Figure 12. If the distance between antennas is increased twice, the input power has to be increased by 5 dBm in order to obtain the same voltage level at the RF rectifier output. The minimum input power needed for a reliable start-up of the self-powered CP system with the minimum load current (4 µA) is 12 dBm and 16 dBm for 3 mm and 6 mm distance between the external and on-chip antennas is required, respectively. The regulated output voltage from the self-powered CP system was set to 0.4 V, and we can observe from Figure 12 that the system can reliably regulate the output voltage to the required value with the accuracy of ±1.25%. Naturally, with higher input power, one can achieve a higher value of the output load current. Regarding the CP system itself, the efficiency was not influenced. On the other hand, the efficiency of the overall CP system including the RF energy harvester will be significantly less than 1%. Such a low efficiency is primarily associated with the quality factor of the on-chip inductor and with the effective chip area in addition to degrading effects (described in [31]).

## 5. Discussion and Conclusions

From the performed analysis, it has been shown that the proposed concept of RF-EH application using the CP-based DC-DC as a PMU can achieve promising performance even though it is designed with standard pure CMOS technology. In order to highlight the important features of the proposed wireless power transfer concept, the developed CP-based DC-DC converter was compared to other state-of-the-art works (Table 1), where the results obtained from measurement under typical conditions are presented. From Table 1, one can observe that the developed DC-DC converter is able to work with the similar input voltage values as reported in other works, except for designs presented in [40,41,42,43,44]. On the other hand, in our work, the converter was designed for ultra-low voltage ICs; therefore, the output voltage was much lower than 1 V. The maximum peak current of 38 µA was achieved for the input voltage of 0.2 V. If the input voltage increased, the output current of more than 100 µA can be reached. The power conversion efficiency of the proposed converter is better than the one reported in the state-of-the art works, except for [40,41,45]. However, the converters presented in [40,41] are designed for higher values of the output voltage. Finally, it is important to underline that the proposed topology achieves the best efficiency compared to the works without any MPPT algorithm [24,44,46,47,48]. Therefore, one can assume that the overall efficiency of the designed converter might be improved by using a proper MPPT algorithm. Additionally, many converters are unregulated and the power consumption of auxiliary circuits forming the feedback loop are not considered in these cases. Moreover, in the proposed design, the power throughput is extended to cover a broader range of applications. On the other hand, our solution needs bulky external components and it is not currently optimized for full integration. The presented results, proved by measurement of prototype chips, demonstrate the suitability of the proposed DC-DC converter for applications in low-power and low-voltage integrated systems. Additionally, it was shown that the developed converter can be conveniently used in the on-chip RF-based energy harvester as a wireless power transfer system. This application concept was verified by measurements, and we do believe that its implementation in a standard pure CMOS technology renders the performed research even more valuable. Finally, it is important to note that various energy harvesters can be used as the input source for the proposed charge pump system. Although in our case the RF harvester was used, there is no limitation for using a different input energy source with an appropriate voltage range. The only strict requirement is that the minimum input voltage must be about 200 mV to ensure the proper functionality of all blocks in the proposed CP system. Since the proposed CP system was designed using bulk-driven technique, the maximum input voltage is limited to the value of 300 mV. On the other hand, we have to underline that the proposed CP system was designed for low-power and low-voltage applications. Therefore, using the energy harvesters generating relatively higher voltages at the output is rather limited or requires additional circuitry in order to reduce the output voltage to the appropriate level.

## Figures and Tables

**Table 1:** Comparison of achieved results relative to other state-of-the-art works.

		This Work	[46]	[47]	[40]	[41]	[42]	[43]	[48]	[44]	[45]	[24]
**Year**		2021	2018	2020	2019	2017	2017	2016	2012	2015	2020	2020
**Process node**	(nm)	130	65	7	65	180	130	180	65	180	65	28
**Area**	($10^{3}\;\mu m^{2}$)	543	32	-	470	552	835	1000	783	48	1400	11.6
**V_IN_**	(V)	0.18–0.3	0.1–0.3	0.15	>0.55	0.5–1.8	0.25–1	0.45–3	0.12–0.16	0.5–1	0.25–1	0.04–0.1
**V_OUT_**	(V)	0.4–0.6	1.2 ^(1)^	0.63	1.8–2.5	1.8	1	3.3	0.77–1.32	1.8	0.9–1.5	-
**V_OUT_ ripple**	(mV)	27.9	0.1–4	20	18	76	80	-	-	-	-	-
**Peak I_OUT_**	(μA)	38 ^(1)^	5 ^(1)^	1	35	19.5	500	15	<12	<7	-	-
**P_OUT_**	(μW)	15.2 ^(1)^	6 ^(1)^	0.68	35–70	<35.1	<500	<50	<10	10.8 ^(2)^	1–100	2 ^(4)^
**Peak η**	(%)	43 ^(3)^	45 ^(3)^	31.76 ^(3)^	70.8	60–72	43 ^(3)^	81	40 ^(3)^	52 ^(3)^	>80 ^(5)^	38.9 ^(3)^
**F_SW_**	(MHz)	0.05 (extern)	15.2	4	1	0.1	<4.25	-	<20	-	0.1–2	1
**Topology**		BD cross-coupled	Cross-coupled	-	Series-parallel	-	Dickson	-	-	-	Boost+Buck SCPC	Hybrid
**Number of stages**		Multi-branch (3 per branch)	3	32	2	2	6	-	10	3	-	2
**MPPT/Regulation**		N/Y	N/N	N/Y	Y/Y	2D/Y	3D/Y	2D/Y	N/N	N/Y	2D/Y	N/N
$FOM_{START-UP}$ **^(6)^**		0.0952 ^(7)^	0.8512	0.2372	-	-	0.0172	0.1248 ^(8)^	-	-	-	0.0648 ^(9)^

^(1)^ @ $V_{IN}$ = 0.2 V; ^(2)^ @ $V_{IN}$ = 0.7 V; ^(3)^ @ $P_{OUT}$ listed; ^(4)^ @ $V_{IN}$ = 0.1 V; ^(5)^ @ $V_{IN}$=0.3 V, $P_{OUT}$ = 5.5 µW; ^(6)^ $FOM_{START-UP}$ = $\frac{C_{LOAD}}{C_{FLY}}\frac{T_{SW}}{t_{START-UP}}\frac{\Delta V_{OUT}}{V_{IN}}M$, where *M* represent voltage conversion ratio; ^(7)^ @ $R_{LOAD}$ = 476 kΩ, $V_{IN}$ = 200 mV, $\Delta V_{OUT}$ = 230 mV, (170 mV → 400 mV), where 170 mV is minimum start-up voltage restricted by comparator supply voltage requirement; ^(8)^ @ $V_{IN}$ = 2.1 V; ^(9)^ Only open circuit time response is considered.

## Data Availability

Not applicable.
